# Study protocol of REGOSARC trial: activity and safety of regorafenib in advanced soft tissue sarcoma: a multinational, randomized, placebo-controlled, phase II trial

**DOI:** 10.1186/s12885-015-1143-y

**Published:** 2015-03-14

**Authors:** Thomas Brodowicz, Bernadette Liegl-Atzwager, Emmanuelle Tresch, Sophie Taieb, Andrew Kramar, Viktor Gruenwald, Marie Vanseymortier, Stéphanie Clisant, Jean-Yves Blay, Axel Le Cesne, Nicolas Penel

**Affiliations:** 1 Comprehensive Cancer Center Vienna – MusculoSkeletal Tumors, Medical University Vienna – General Hospital, Vienna, Austria; 2 Comprehensive Cancer Center Graz – Subunit Sarcoma, Institute of Pathology, Medical University Graz, Graz, Austria; 3 Biostatistics Unit, Centre Oscar Lambret, Lille, France; 4 Medical Imaging, Centre Oscar Lambret, Lille, France; 5 SIRIC OncoLille, Lille, France; 6 Medizinische Hochschule Hannover, Hannover, Germany; 7 Clinical Reserch Unit, Centre Oscar Lambret, Lille, France; 8 Medical Oncology, Centre Léon Bérard, Lyon, France; 9 Medical Oncology, Gustave Roussy, Villejuif, France; 10 Medical Oncology, Centre Oscar Lambret, Lille, France

**Keywords:** Angiogenesis, Placebo-controlled trial, Progression-free survival, Randomized phase II trial, Regorafenib, Sarcoma

## Abstract

**Background:**

Angiogenesis, among other signaling pathways, plays a key-role in sarcoma biology. Regorafenib (RE) has recently been shown to be effective in imatinib and sunitinib-refractory GIST in a phase III trial.

**Methods/design:**

We are conducting an international trial (France, Austria and Germany) consisting in 4 parallel double-blind placebo-controlled randomized (1/1) phase II trials to assess the activity and safety of RE in doxorubicin-refractory STS (ClinicalTrials.gov: NCT01900743). Each phase II trial is dedicated to one of the 4 following histological subgroups: liposarcoma, leiomyosarcoma, synovial sarcoma and other sarcoma. Within each randomized trial the following stratification factors will be applied: countries and prior exposure to pazopanib. Key-eligibility criteria are: measurable disease, age ≥18, not > 3 previous systemic treatment lines for metastatic disease, metastatic disease not amenable to surgical resection. The primary endpoint is progression-free survival (PFS) according to central radiological review. Secondary endpoints are: Toxicity (NCI-CTC AE V4.0); time to progression; Growth modulation index in pts receiving RE after randomization; 3 and 6 months PFS-Rates, best response rate and overall survival. Each phase II trial will be separately analyzed. In 3 trials, statistical assumptions are: PFS0 = 1.6 & PFS1 = 4.6 months; 1-sided α = 0.1; β = 0.05 with a total sample size of 192 pts. To take into account the rarity of synovial sarcoma, the statistical assumptions are: PFS0 = 1.6 & PFS1 = 4.6 months; 1-sided α = 0.1; β = 0.2 Tumor assessment is done monthly during the 4 first months, and every 3 months thereafter. After central radiological confirmation of tumor progression, an optional open-label option is offered to eligible patients.

**Discussion:**

The design of this trial allows an assessment of regorafenib activity over placebo in four sarcoma strata and might provide evidence for launching a phase III trial. This study includes both integrative and exploratory translational research program. The study is enrolling since June 2013 (Trial Registration Number: EudraCT N°: 2012-005743-24, on the 15^th^ February 2012).

## Background

### Clinical setting

Soft tissue sarcomas (STS) are a heterogeneous group of tumor, accounting for at least 2% of adult cancers. Soft tissue sarcoma comprises more than 50 different histological subtypes. The 4 major subgroups are: liposarcoma, leiomyosarcoma, synovial sarcoma and other sarcomas. Despite large en bloc resection plus radiotherapy more than 40% of patients experience metastatic recurrence. For patients with advanced disease, palliative chemotherapy based on doxorubicin (+/- ifosfamide) represents the standard of care. Doxorubicin provides a response rate of about 20% and a median overall survival of about 12-18 months [1,2]. Today, there is no consensual treatment after intolerance or failure of doxorubicin. Nevertheless, some new drugs provide promising signs of activity (trabectedin, gemcitabine-docetaxel, pazopanib, eribuline … [2-5]), but until now, none of them could be considered as a standard of care after doxorubicin-failure or intolerance. Main subtypes of soft tissue sarcoma are: liposarcoma (25-30%), leiomyosarcome (25-30%) and synovial sarcomas (10%). Angiogenesis is of crucial importance for growth and dissemination of malignancies. In this process vascular endothelial growth factors and other pro-angiogenic factors are of major importance. There is a large body of evidence that angiogenesis plays a key-role in the development of sarcomas [6-13].

Moreover, one of the promising drugs for the treatment of STSs, pazopanib is an oral angiogenesis inhibitor with activity against vascular endothelial growth factor receptors (VEGFR) 1, 2 and 3, and platelet-derived growth factor receptor (PDGFR) [5]. Excluding liposarcomas, pazopanib improves the PFS over placebo [14].

### Investigational treatment

Regorafenib (BAY 73-4506) is an orally bioavailable multikinase inhibitor targeting tumor cells, vasculature, and the tumor microenvironment. Regorafenib (BAY 73-4506) binds to and inhibits VEGFR-1, - 2 and -3, and tumor cell signaling kinases (RET, KIT, PDGFR, and Raf), which may result in the inhibition of tumor angiogenesis and tumor cell proliferation. Regorafenib (BAY 73-4506) shows potent, oral activity in a wide variety of preclinical xenograft models. Regorafenib has completed a first set of phase I- III clinical trials [15,16]. In the phase I trial, one of the three responding patients had had an advanced sarcoma [15].

### Prior experience with regorafenib

Regorafenib showed efficacy and manageable toxicity in the treatment of refractory colorectal cancers (CRC) and GIST in two phase III trials.

The CORRECT study was an international, multicenter, randomized, double-blind, placebo-controlled Phase III study that enrolled 760 patients with mCRC whose disease had progressed during or within 3 months following last administration of approved standard therapies, which included a fluoropyrimidine, oxaliplatin, irinotecan, bevacizumab and cetuximab or panitumumab. Patients who had withdrawn from standard treatment due to unacceptable toxicity warranting discontinuation of treatment and precluding retreatment with the same agent prior to progression of disease were also allowed into the study. Patients were randomized to receive either regorafenib plus best supportive care (BSC) or placebo plus BSC. Treatment cycles consisted of 160 mg of regorafenib (or matching placebo) once daily for three weeks on/one week off. The study met its primary endpoint, showing statistically significant improvement in overall survival (OS) by 29% (HR = 0.77, p = 0.0052, median OS: 6.4 months vs. 5.0 months for the placebo group) in patients with metastatic colorectal cancer (mCRC) whose disease had progressed after approved standard therapies. Additionally, findings from the secondary endpoints of the CORRECT study showed statistically significant improvement in progression-free survival (PFS) (HR = 0.49, p < 0.000001, median PFS: 1.9 months vs. 1.7 months) and an improvement in disease control rate (44.8% vs. 15.3%) in patients treated with regorafenib compared to those treated with placebo. The most common drug-related, treatment-emergent adverse events included fatigue (47.4% vs. 28.1%), hand-foot skin reaction (46.6% vs. 7.5%), diarrhea (33.8% vs. 8.3%), anorexia (30.4% vs. 15.4%), hypertension (27.8% vs. 5.9%), oral mucositis (27.2% vs. 3.6%) and rash/desquamation (26.0% vs. 4.0%) for patients receiving regorafenib as compared to placebo [17]. Regarding these findings, regorafenib is now approved for the treatment of mCRC in USA, Europe, and many other countries.

GRID was a randomized, double-blind, placebo-controlled, multi-center, Phase III study of regorafenib for the treatment of GIST. It enrolled 199 patients whose disease had progressed despite prior treatment with imatinib and sunitinib. Patients were randomized in a 2:1 ratio to receive either regorafenib (160 mg once daily, three weeks on/one week off) plus BSC or placebo plus BSC to evaluate efficacy and safety. The primary endpoint of this trial was PFS, and secondary endpoints included overall survival, time to progression, disease control rate, tumor response rate, and duration of response. The GRID study met its primary endpoint of progression-free survival (PFS) (HR = 0.27, p < 0.0001). The median PFS was 4.8 months in the regorafenib arm vs. 0.9 months in the placebo arm. The most common drug-related, treatment-emergent adverse events (occurring in at least 10% of patients during double-blind treatment) included hand-foot skin reaction (56.1% vs.15.2%), hypertension (48.5% vs. 16.7%), diarrhea (40.9% vs.7.6%), fatigue (38.6% vs. 27.3%), oral mucositis (37.9% vs. 9.1%), alopecia (23.5% vs. 3.0%), hoarseness (22.0% vs. 4.5%), anorexia (20.5% vs. 7.6%), maculopapular rash (18.2% vs. 3.0%), nausea (15.9% vs. 9.1%), constipation (15.2% vs. 7.6%), myalgia (13.6% vs. 9.1%), and voice alteration (11.4% vs. 3.0%) for patients receiving regorafenib as compared to placebo [18]. Regarding this findings regorafenib in now approved for the treatment of GIST in USA and European countries.

### Rationale for the study

The standard of care for metastatic STSs is doxorubicin +/- ifosfamide. After failure or intolerance to doxorubicin, there is no standard of care. In Europe, two drugs are currently approved for the treatment of soft tissue sarcoma after failure/intolerance to doxorubicin: trabectedin (Yondelis ®) for all histological subtype and pazopanib (Votrient ®) for all subtypes excluding liposarcomas. Trabectedin is mostly active in liposarcoma and leiomyosarcoma. Pazopanib is active in non-lipomatous sarcomas. New treatments are needed for the various histological STS subtypes; an unmet medical need so far.

The study population is represented by patients with metastatic STS having received at least doxorubicin (or other anthracyclin) as a previous therapy line. Patients will have measurable disease by Response Evaluation Criteria in Solid Tumors (RECIST 1.1) and will have documented disease progression according to RECIST within the last 6 months before entry in the study, after treatment with doxorubicin (or other anthracyclin derivatives). The study consists of 4 parallel randomized phase II trials, defined by the 4 following histological subgroups: liposarcoma, leiomyosarcoma, synovial sarcoma and other sarcomas (according to local histology).

## Methods/Design

### Study objectives

The primary objective of the trial is to investigate, in each of the 4 parallel studies, whether treatment with regorafenib improves progression-free survival as compared to placebo.

The secondary objectives include other efficacy outcomes and an evaluation of the tolerance/toxicity of regorafenib in the study population. A translational program research is part of the study (see below).

### Study endpoints

The primary endpoint of this phase II study is progression-free survival (PFS) according to modified Response Evaluation Criteria in Solid Tumors (RECIST 1.1) with central radiological review. Progression-Free Survival will be measured from the date of randomization until the date of radiological progression or death of any cause (whatever occurs first). Patients without tumor progression or death at the time of analysis will be censored at their last date of radiological tumor assessment. The date of disease of progression will be the date of first observation of progression (primary analysis on intent-to-treat analysis, according to RECIST 1.1 guidelines and central radiological review).

The secondary endpoints are the following:

- Disease Control Rate (DCR),
  Disease Control Rate is defined as the proportion of patients who have a best response rating of complete response (CR), partial response (PR) or stable disease (SD) according to RECIST guidelines 1.1 that is achieved during treatment or within 30 days after termination of study medication. Stable disease must be at least 6 weeks in duration.
- Time To Progression (TTP),
  Time to progression will be measured from the date of randomization to the date of the first progression. Patients who die from causes other than progression are censored at the date of death.
- Tumor Response Rate (RR)
  Tumor Response Rate is defined as the proportion of patients with the best overall tumor response of partial response (PR) or complete response (CR) according to RECIST 1.1 guidelines that is achieved during treatment or within 30 days after termination of study medication.
- Duration of response,
  Duration of response is measured from complete or partial response to progression or death.
- Overall survival (OS),
  Overall Survival is measured from the date of randomization until the date of death due to any cause.
- Growth Modulation Index (GMI)
  The Growth modulation index is defined as the ratio of time to progression under regorafenib to time to progression under previous treatment. The growth modulation index will be explored in patients receiving regorafenib after randomization [18].
- Toxicity
  Toxicity will be evaluated according to NCI-CTC AE V4.0

### Overview of the study design

This is an international trial consisting of 4 parallel randomized, double-blind, placebo-controlled, multi-center phase II studies to evaluate the efficacy and safety of regorafenib in patients with histologically proven metastatic and/or unresectable STS after failure or intolerance to doxorubicin (or other anthracyclin). Patients must have shown objective disease progression at study entry.

**Figure 1:**
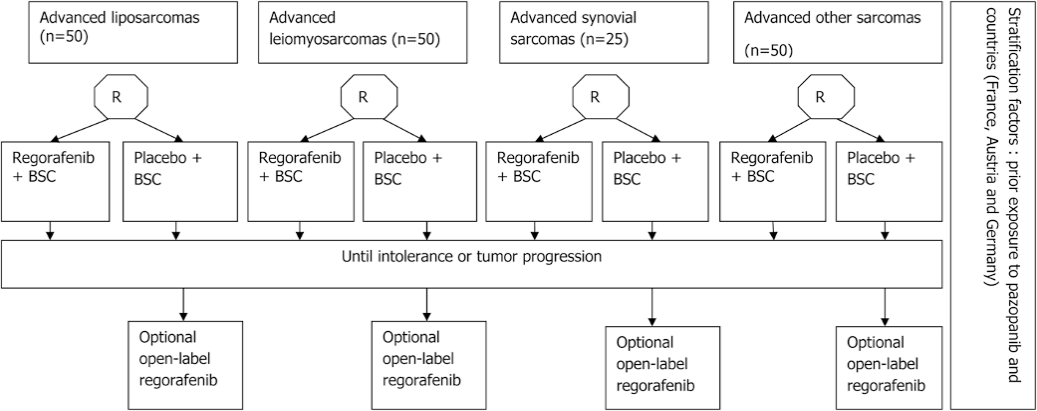
**Trial Design: 4 parallel phase II double-blind placebo-controlled phase II trials.**

Patients will be registered at the Clinical Research Unit of the Oscar Lambret Cancer Center prior to start the treatment, and after verification of eligibility criteria. Patients will be randomized to receive oral regorafenib or placebo in a 1:1 ratio, until disease progression (RECIST 1.1 guidelines), death, unacceptable toxicity or withdrawal of consent for any reasons. Patients receiving placebo who experience disease progression may be offered open-label regorafenib after checking of eligibility criteria and real-time central radiological review of imaging to confirm progression according to RECIST 1.1 (Figure 1).

An independent data monitoring committee is planned to assess the risk/benefit ratio after enrollment of the 50 first patients.

The study is composed of 3 periods:

- Screening Period
- Treatment Period during which either regorafenib or placebo will be administered (The Treatment Period includes an end of treatment visit and a 30 (+/-7) day follow-up period that ends with a safety follow-up visit).
- Survival Follow-up Period during which survival status will be monitored

During the Screening Period and the Treatment Period, patients are considered “on-study”; during the Survival Follow-up Period, patients are considered “off-study”. During the treatment period the tumour assessment will be done every month during the 4 first months and then every 3 months. Study assessments is summarized in Table 1 (Flow-chart).

**Table 1:** **Study procedures and flow-chart**

	Screening 14 days	d1, d30, d60, d90, d120	After the 4^th^month, every 3 months until tolerance or progession
Physical exam	X	X	X
Safety	X	X	X
Availbility of tumour sample	X		
Thoracic and abdominopelvic CT-Scann	X	X	X
Echocardio or MUGA	X		X
Hematology INR TCA	X	X	X
Biochemistry (1)	X	X	X
Urine Dipstick	X	X	X
Serum sample for TR programm		X	

(1) Serum creatinine, Glomerular filtration rate (Cockroff and Gault), AST, ALT, Bilirubin, Alkaline phosphatases, Amylase and lipase, CPK.

The patients will be randomly allocated to one of the treatment described above.

- Regorafenib: 4 tablets, once daily, 3 weeks on/1 week off

+ Best Supportive Care

Or

- Placebo: 4 tablets, once daily, 3 weeks on/1 week off

+ Best Supportive Care

until progression or unacceptable toxicity in both arms.

Best supportive care includes any method to preserve the comfort and dignity of the patients and excludes any disease-specific anti-neoplastic agent.

Details on dose-adaptations, prohibited concomitant medications and the study flow-chart could be obtained by request to the corresponding author.

### Eligibility criteria

All the following must be met at the time of screening.

1. Age ≥18 years of age
2. Histological documentation of soft tissue (including uterus) sarcoma with available FFPE blocks obtained.
3. Prior treatment with doxorubicin or other anthracyclin
4. Metastatic disease not amenable to surgical resection with curative intent
5. Documentation of progression before study entry
6. Measurable disease, defined as at least 1 unidimensionally measurable lesion on a CT scan as defined by RECIST 1.1.
7. Eastern Cooperative Oncology Group (ECOG) performance status ≤1
8. Life expectancy of at least 3 months
9. Adequate bone marrow, renal, and hepatic function, as evidenced by the following within 7 days of study treatment initiation:
  - Absolute neutrophil count (ANC) ≥1,500/mm3
  - Platelets ≥100,000/mm3
  - Hemoglobin ≥9.0 g/dL
  - Serum creatinine ≤1.5 x upper limit of normal (ULN)
  - Glomerular filtration rate (GFR) ≥30 ml/min/1.73 m2
  - AST and ALT ≤2.5 x ULN (≤5.0 × ULN for patients with liver involvement of their cancer
  - Bilirubin ≤1.5 X ULN
  - Alkaline phosphatase ≤2.5 x ULN (≤5 x ULN with liver involvement of their cancer)
  - Amylase or lipase ≤1.5 x ULN
  - Spot urine must not show 1+ or more protein in urine or the patient will require a repeat urine analysis. If repeat urine analysis shows 1+ protein or more, a 24-hour urine collection will be required and must show total protein excretion <1000 mg/24 hours
10. INR/PTT ≤1.5 x ULN - Patients who are therapeutically treated with an agent such as warfarin or heparin will be allowed to participate provided that no prior evidence of underlying abnormality in coagulation parameters exists. Close monitoring of at least weekly evaluations will be performed until INR/PTT is stable based on a measurement that is pre-dose as defined by the local standard of care
11. Women of childbearing potential and male patients must agree to use adequate contraception for the duration of study participation and up to 3 months following completion of therapy. Adequate contraception is defined as any medically recommended method (or combination of methods) as per standard of care.
12. Recovery to National Cancer Institute-Common Terminology Criteria for Adverse Events (NCI-CTCAE) v4.0 Grade 0 or 1 level or recovery to baseline preceding the prior treatment from any previous drug/procedure related toxicity (except alopecia, anemia, and hypothyroidism).
13. In the assessment of the investigator, patient is able to comply with study requirements
14. Signed, IRB-approved written informed consent as approved by ethical and regulatory committee: French Ethical Committee (“*Comité de Protection des Patients Nord-Ouest IV”*; date of approval 21th March 2013), and Austrian Ethical Committee (“Ethik Kommission Medizinische Universität Wien (n° 1376/2013)) and French Drug Agency (“*Agence Nationale de Sécruité du Médicament*”; date of Approval 8^th^ March 2013).

#### Exclusion criteria

Patients who meet any of the following criteria at the time of screening will be excluded from the study.

1. More than 3 lines of systemic treatment for metastatic sarcoma
2. Some particular histologies: GIST, osseous sarcoma, embryonnal or alveolar rhabdomyosarcoma)
3. Primary bone sarcoma
4. Prior treatment with regorafenib
5. Known history of or concomitant malignancy likely to affect life expectancy in the judgment of the investigator
6. Pregnant or breastfeeding patients. Women of childbearing potential must have a pregnancy test performed a maximum of 7 days before start of treatment
7. Major surgical procedure, open biopsy, or significant traumatic injury within 28 days before start of Day 1 of treatment
8. Active cardiac disease including any of the following: Congestive heart failure (New York Heart Association [NYHA]) ≥ Class 2, Unstable angina (angina symptoms at rest), new-onset angina (begun within the last 3 months), Cardiac arrhythmias requiring anti-arrhythmic therapy (beta blockers or digoxin are permitted)
9. Uncontrolled hypertension. (Systolic blood pressure >150 mmHg or diastolic pressure >90 mmHg despite optimal medical management)
10. Arterial or venous thrombotic or embolic events such as cerebrovascular accident (including transient ischemic attacks), deep vein thrombosis, or pulmonary embolism
11. Ongoing infection > Grade 2 according to NCI Common Terminology Criteria for Adverse Events version 4.0 (CTCAE v. 4.0)
12. Known history of human immunodeficiency virus (HIV) infection
13. Known history of chronic hepatitis B or C
14. Patients with seizure disorder requiring medication
15. History of organ allograft
16. Evidence or history of bleeding diathesis. Any hemorrhage or bleeding event > Grade 4 within 4 weeks of start of treatment
17. Non-healing wound, ulcer, or bone fracture
18. Renal failure requiring hemo- or peritoneal dialysis
19. Dehydration according to NCI-CTC v 4.0 Grade >1
20. Substance abuse, medical, psychological, or social conditions that may interfere with the patient’s participation in the study or evaluation of the study results
21. Known hypersensitivity to any of the study drugs, study drug classes, or excipients in the formulation including lactose
22. Interstitial lung disease with ongoing signs and symptoms at the time of informed consent
23. Inability to swallow oral medications, Any malabsorption condition
24. Pleural effusion or ascites that causes respiratory compromise (Grade 2 dyspnea)
25. Unwilling to provide consent for genetic studies of tumor, whole blood, or plasma specimens

### Statistical considerations

#### Statistical hypothesis – Sample size calculation

The primary endpoint is progression-free survival (PFS) according to RECIST 1.1 guidelines and with central radiological review. The sample size is calculated on the basis of the primary endpoint. The study consists of 4 parallel phase II trials in 4 sub-populations defined by histology: liposarcoma, leiomyosarcoma, synovial sarcoma and other sarcomas. Recent literature data suggest that PFS with placebo is about 1.6 months [14]. The expected PFS with regorafenib is 4.6 months. Statistical assumptions for the 3 most frequent histological subtypes (liposarcoma, leiomyosarcoma and other sarcomas) are: PFS0 = 1.6, PFS1 = 4.6, alpha = 0.1 (one-sided) and power (1-β) = 0.95, the sample size is 50 patients per stratum (30 expected events). For the Synovial sarcoma stratum, based on its low prevalence and in order to not delay significantly the duration of the study, lower power is considered acceptable and only 25 patients will randomized in this cohort (Alpha = 0.10, Beta = 0.20, 16 events and 25 patients).

At the end, the total number of patients is calculated as follows: (50x3) + 25 = 176 (+10% of non-valuable patients: 192)

#### Randomization and stratification

Patients will be centrally randomized to receive regorafenib or placebo, in a double blind fashion, and in a 1:1 ratio respectively. Four strata will be identified: leiomyosarcoma (50 patients), liposarcoma (50 patients), other sarcoma (50 patients) and synovial sarcoma (26 patients). A permuted blocks randomization technique will be used for treatment allocation. Within the 4 strata, stratification factors will be: prior exposure to pazopanib (yes/no) and countries.

#### Analysis sets

The following patient populations will be considered in the final analyses.

- Intention-to-treat population: All randomized patients will be analyzed in the arm they were allocated by randomization.
- Per protocol population: All patients who are eligible and have started their allocated treatment (at least one dose of the study drug)
- Safety population: All patients who have started treatment (at least one dose of the study drug)

A patient will be considered to be eligible if he/she did not have any major deviations from the patient entry criteria listed in chapter 3 of the protocol. Eligibility will be assessed by the Study Coordinator based on the review of each patient file.

The primary analysis will be conducted on the Intention-to-treat population.

#### Statistical analysis

Progression free survival will be analyzed in the intent to treat population. For the primary analysis, in each stratum, a one sided logrank test stratified for pre-specified stratification factors will be used, and tested at the significance level of 0.10. The size of the treatment difference will be measured by the estimated hazard ratio and its 95% confidence interval.

The progression free survival rates will be estimated as a function of time by the Kaplan-Meier method. Overall survival will be analyzed in the intent to treat population. The overall survival rates will be estimated as a function of time by the Kaplan-Meier method. Time to progression rates will be estimated as a function of time by the Kaplan-Meier method in the intent to treat population. Response rates at 3 and 6 months, progression-free rates at 3 and 6 months will be analyzed by descriptive techniques (intent to treat analysis).

The occurrence of adverse events will be analyzed in the safety population, by descriptive techniques. For each type of adverse event, the worst grade observed across the whole therapy will be tabulated by treatment arm, and the percentages of grade 2+ and grade 3+ cases will be provided. For events occurring in more than 10% of the cases at a grade 2+, the cumulative incidence will be computed as a function of time for each grade, by treatment arm, considering discontinuation of therapy for reasons other than an adverse event as a competing risk.

#### Pre-planned sensitivity or exploratory analyses

A sensitivity analysis of progression free survival will be conducted in the per protocol population, if more than 5% of the randomized patients are excluded from the analysis.

Data from patients treated with regorafenib after “cross-over” will be analyzed (activity – PFS, Time to progression, best objective response, PFR3 and PFR6, OS- and tolerance) with classical descriptive methods.

### Translational research (TR) program

TR analyses will be done at the Institute of Pathology Medical University Graz in Austria.

The analysis consists of two parts:

1. The first part “integrated TR” includes the central review of histopathology on paraffin embedded tumor blocks. The central confirmation of histopathological diagnoses is mandatory to include patients into the outlined study. Immunohistochemistry (IHC) and molecular analysis (FISH and RT-PCR) will be performed to confirm the diagnosis, if not previously performed in reference centers.

2. The second part “exploratory TR” component will further characterize the nature of the genetic change by exploring the mutational status of the tumor samples using the Ion AmpliSeq™ Cancer Panel, Life Technologies Corporation. In addition it is planned to construct a tissue-micro arrays (TMA) from FFPE material to allow a large-scale evaluation of molecular aberrations and downstream effects on pathway activation.

The key objectives of this translational research are:

- Identification and characterization of biomarkers.
- Exploration of specific molecular changes that can potentially be used as predictive markers of response to regorafinib.
- Better definition of the patient population most sensitive to regorafinib.

Formalin fixed, paraffin embedded (FFPE) or fresh frozen tissue samples collected either from the primary tumor or from metastatic sites, or both will be analyzed.

Immunohistochemistry and molecular analysis [fluorescence in situ hybridisation (FISH) and reverse transcriptase polymerase chain reaction (RT-PCR)], will be performed to investigate chromosomal aberrations characteristic for specific sarcoma subtypes (e.g. Synovial Sarcoma SYT-SSX1 and SYTSSX2). Genetic changes will be investigated by exploring the mutational status of the tumor samples using the Ion AmpliSeq™ Cancer Panel V2 SNP analysis (47 genes, 790 hotspots), Life Technologies Corporation. In addition to this screening approach the full coding sequence of VEGFR1-3, TIE2, PDGFRB, FGFR1, KIT, RET1 and RAF will be explored. Submitted paraffin blocks will be used to construct a tissue microarray (TMA). This TMA will allow exploring potential predictive or prognostic factors for treatment response and eventually validation of newly discovered genes as diagnostic and therapeutic targets. The panel of IHC antibodies will strongly depend on the results of third generation sequencing. Formalin fixed paraffin embedded (FFPE) tumor blocks will be collected for all patients (this is mandatory for study participation). Blocks must be accompanied by electronic information on histopathology reports and, if applicable, by written reports on previously performed molecular analysis of the tumor. Slides are not acceptable. FFPE blocks may be from primary or metastatic sites. Residual FFPE material will be used for the planed exploratory translational research. FFPE materials must be from tissue samples taken prior to any treatment with regorafinib. Tumor blocks will be return to the patient center after analysis.

#### Fresh frozen tissue samples (optional)

The collection of fresh frozen tissue samples (from primary or metastatic sites) is optional for this study. All fresh frozen samples (including any samples from re-biopsy) must be taken prior to any treatment with regorafinib.

Workflow at the Institute of Pathology Medical University Graz, Austria.

Representative paraffin blocks of 192 soft tissue sarcomas will be submitted to the Institute of Pathology. The following information is mandatory: patients’ gender, age, tumor location, specification primary tumor or metastases. The pathology report will be anonymized and enclosed including the diagnosis, IHC profile and result of molecular diagnostics if available.

Working steps:

1. Every sample will get an internal examination number.
2. One HE slide will be cut from every paraffin block for evaluation of tumor tissue (tumor tissue will be marked and the amount of viable tumor will be given by the pathologist). If the diagnosis has been confirmed by IHC and Molecular Diagnostics (FISH or RT-PCR) these analyses will not be repeated. If this information is not available the analyses will be performed in Graz.
3. The paraffin blocks will be cut to extract DNA and RNA for third generation sequencing (see below).
4. Three to five tissue cores will be taken from every paraffin block to conduct a TMA.

3rd generation sequencing: Extended Cancer Panel Analysis of 192 sarcoma samples:

Genetic changes will be investigated by exploring the mutational status of the tumor samples using the Ion AmpliSeq™ Cancer Panel V2 SNP analysis (47 genes, 790 hotspots) and in addition the full coding sequence of VEGFR1-3, TIE2, PDGFRB, FGFR1, KIT, RET1, RAF will be explored.

The analysis will be based on SNP/InDel calling for all samples.

Tissue in the paraffin block is cut with a scalpel along the border of normal to tumor tissue.

Sections are prepared from the FFPE block. Tumor tissue and normal tissue (if available) will be separately collected. DNA and RNA are extracted from the tumor samples using the Qiagen Allprep kit. DNA is quantified by Picogreen fluorescence and concentration is normalized. Each DNA is amplified with the respective primer sets, products are quantified, combined and one library is constructed per tumor sample. 5 libraries are sequenced together on a 318 chip (~3-4Mio reads) to obtain an average 2000× coverage of each amplicon. FISH, RT-PCR and IHC will be performed according to standard techniques.

## Discussion

### Accrual

**Figure 2:**
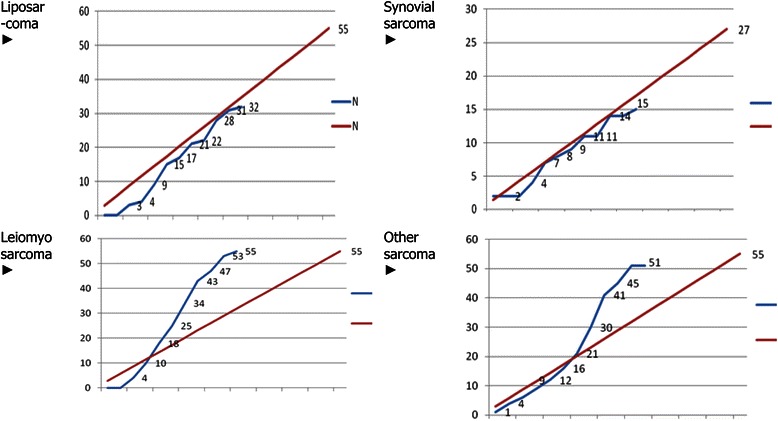
**Accrual at the date of 15 May 2014 (blue lines: current accrual/ red lines: theorical accrual).**

The study is enrolling since June 2013 (at the time of the preparation of this paper in 26 sites in France and Austria) The accrual is faster than expected. Per 1^st^ of May 2014, 2 strata are already closed to recruitment (leiomyosarcomas and other sarcomas). The number of enrolled patients is 152 (out of 192). The recruitment is still ongoing for synovial sarcoma and liposarcoma strata. According to the recruitment rate, the study will be definitely closed to recruitment between December 2014 and February 2015 (Figure 2).

### Real-time central radiological reading

One of the main issues of the trial management is about the real-time central radiological reading for confirmation of progression according to RECIST with subsequent unblinding before open-label treatment with regorafenib. Ethical committees and investigators are concerned about the concept of placebo-controlled trial. To make sure that this study is not detrimental to the patients, we have proposed to perform tumor assessment every month during the 4 months. According to previously published trial [14], median time to progression is expected to be about 1.6 months in patients receiving placebo. This implies to increase the number of CT-scan assessments at the beginning of study. As soon as the progression is confirmed by central radiological review, patients who received placebo are offered to receive regorafenib on an open-label basis. Patients receiving regorafenib are off-study (treatment with regorafenib beyond disease progression could be discussed case by case with the sponsor). This process is time- and resource-consuming; nevertheless it is regarded to be feasible even within a multinational study. Radiological central review and unblinding could be done within 48 hours after having received CT-Scan copies (baseline and CT with suspected disease progression).

### Methodological discussion

The writing committee had faced to 2 major issues: (i) the vast heterogeneity of STS in terms of histologies and prior treatments, (ii) the absence of standard of care in this setting and (iii) the choice of the primary endpoint. The proposed trial design is a complex one, integrating randomization, stratification and the application of 4 parallel placebo-controlled randomized phase II trials. This could be discussed point by point.

Most of phase II trials assessing the activity of a new drug in STS are not randomized. There at least 2 reasons justifying the randomization: the choice of placebo as internal comparator and the choice of the primary endpoint. Non-randomized phase II trials are exposed to selection biases related to the respective eligibility criteria. Thus, the use of an internal comparator is helpful to ensure the representativity of enrolled patients. The major issue here is the choice of the comparator. The list of potential comparators is vast, including dacarbazine, gemcitabine, pazopanib, trabectedin … There is no consensual treatment. To avoid never-ending discussion about the optimal choice of the comparator (including “physician choice”), we have decided to use placebo as internal comparator to provide a clear-cut estimate of the drug activity.

Most of phase II trials in STS patients run a fixed-time point primary endpoint (such as 3-month progression-free rate or 6-month progression-free rate). Because tumour shrinkage is rare with such kinds of drugs and especially in case of STS, tumor response is an inadequate primary endpoint. Some other endpoints could be used (Choi criteria, functional imaging such as contrast enhanced ultrasonography or position emission tomography), but none of these criteria is formally validated and standardized in this setting. In this case, we have to measure how the investigational drug slows down the tumor course [19]. As a consequence, because we have used a time-dependent endpoint (PFS), randomization is absolutely necessary. Without randomization, impact on PFS could have been related to the natural history of STS or the drug activity.

Heterogeneity in term of prior management of STS had also to be taken into account. As a consequence, we have used 2 stratification factors in each parallel phase II trials: prior exposure to pazopanib and countries.

Statistical assumption is based on recently published trials [14]. Our trial is a comparative phase II trial, implying a unilateral alpha. There are some debates about comparative versus non-comparative randomized phase II trials [20]. This is largely beyond the topic of this publication.

## Abbreviations

BSC: Best Supportive Care
CRC: Colo-rectal cancer
EORTC: European Organization for Research and Treatment of Cancer
FFPE: Formalin-Fixed, Paraffin-Embedded Tissue
FISH: Fluorescence In situ Hybridization GIST – Gastro-Intestinal Stromal Tumor
IC-50: Half maximal inhibitory concentration
IHC: Immuno-Histo-Chemistry
PFS: Progression Free survival
RECIST: Response Evaluation Criteria In Solid Tumors
RT-PCR: Reverse transcription polymerase chain reaction
SNP: Single-nucleotide polymorphism
STS: Soft Tissue Sarcoma
TMA: Tumor Micro-array
ULN: Upper Limit of Normal
